# The relationship between self-connection and online prosocial behavior among college students: the chain mediating roles of belief in a just world and meaning in life

**DOI:** 10.3389/fpsyg.2026.1864400

**Published:** 2026-06-25

**Authors:** Xia Cai, Xinyu Liu, Xiangcai He

**Affiliations:** 1 School of Psychology, Guizhou Normal University, Guiyang, China; 2 School of Psychology, Chengdu Medical College, Chengdu, China; 3 Research Center for Adolescent Abnormal Psychology and Behavior, Chengdu Medical College, Chengdu, China

**Keywords:** belief in a just world, chain mediation, meaning in life, online prosocial behavior, self-connection

## Abstract

**Objective:**

This study aimed to explore the relationship between self-connection and online prosocial behavior among college students, and to examine the mediating roles of belief in a just world and meaning in life.

**Methods:**

A total of 1,303 college students completed the Self-Connection Scale, the Online Prosocial Behavior Scale, the Belief in a Just World Scale, and the Meaning in Life Scale.

**Results:**

(1) Self-connection positively predicted online prosocial behavior; (2) belief in a just world played a mediating role in the relationship between self-connection and online prosocial behavior; (3) meaning in life played a mediating role in the relationship between self-connection and online prosocial behavior; (4) belief in a just world and meaning in life played a serial mediating role in the relationship between self-connection and online prosocial behavior.

**Conclusion:**

Self-connection not only directly promoted online prosocial behavior among college students, but also exerted indirect effects through belief in a just world and meaning in life. These findings have important implications for fostering online prosocial behavior among college students.

## 1 Introduction

The 55th Statistical Report on Internet Development in China released by China Internet Network Information Center (CNNIC) in 2025 shows that as of December 2024, the number of internet users in China reached 1.108 billion, with an internet penetration rate of 78.6%. With the rapid development and transformation of the internet era, individual behavior patterns have changed significantly. In recent years, negative or antisocial online behaviors such as cyberbullying, cyberharassment, and cyberattacks have been extensively studied (Chen et al., 2017; Kowalski et al., 2014; Zych et al., 2015), whereas online prosocial behavior remains underexplored. Online prosocial behavior refers to voluntary actions conducted in online environments that aim to benefit specific individuals or foster harmonious interpersonal relationships (Erreygers et al., 2018). Online prosocial behavior not only contributes to the development of positive online civilization and serves as an effective counterbalance against cyberattacks, but also enhances social connectivity, improves interpersonal relationship quality, and promotes mental health and self-esteem (Valkenburg and Peter, 2009, 2011). Notably, online prosocial behavior may be a complex phenomenon shaped by multiple coexisting motivations (Amichai-Hamburger, 2008). For example, these include genuine altruism driven by empathy and moral concern; social signaling and impression management aimed at demonstrating moral character and gaining social approval (Bénabou and Tirole, 2006); and norm compliance reflecting participation due to conformity pressure rather than intrinsic motivation (Cialdini and Goldstein, 2004). Because of the anonymity inherent in online environments, the specific motives underlying individuals' online prosocial behavior remain highly uncertain. Therefore, this study does not focus on distinguishing among these motivational subtypes. Additionally, college students are among the most active internet users, exhibiting highly malleable online behaviors. Investigating college students' online prosocial behavior not only advances cyberpsychology research but also contributes to a healthier online ecosystem.

### 1.1 Self-connection and online prosocial behavior

Self-connection refers to the internal state in which individuals maintain alignment with their authentic selves, encompassing three core dimensions: self-awareness, self-acceptance, and self-consistency (Klussman et al., 2022a,b). In recent years, researchers have increasingly begun to focus on the role of self-connection in promoting prosocial behavior. Both self-connection and mindfulness involve awareness and attention to internal states (Klussman et al., 2022a,b), but self-connection extends beyond mere awareness by emphasizing behavioral consistency based on awareness (Zhao et al., 2024). Prior research has found that mindfulness enhances self-connection (Klussman et al., 2022a,b), and mindfulness itself has been demonstrated to promote prosocial behavior (Guo and Chen, 2022). Therefore, self-connection is likely a critical intrapersonal mechanism through which mindfulness influences prosocial behavior. Furthermore, the Self-Determination Theory posits that when individuals establish a connection with their authentic inner selves, their fundamental psychological needs are fulfilled, thereby eliciting autonomous prosocial motivation (Deci and Ryan, 2000). Empirical evidence also indicates that individuals with high self-connection exhibit elevated empathy and altruistic tendencies, enabling them not only to clearly identify their own emotional states but also to accurately infer others' needs based on these states (Krol and Bartz, 2022). However, existing studies predominantly focus on real-world scenarios. With digital technologies deeply embedded in daily life, cyberspace—characterized by unique anonymity, transcendent temporality and spatiality, and symbolic features—has become a pivotal arena for contemporary social interactions. It is also particularly important to explore the role of self-connection in promoting prosocial behavior in network environments. When individuals maintain alignment with their authentic selves online, they are more likely to engage in sincere self-disclosure, thereby establishing deep interpersonal trust and creating a foundation for online prosocial behavior (Bai et al., 2025). Thus, this study proposes the first hypothesis: self-connection can significantly and positively predict online prosocial behavior.

### 1.2 Belief in a just world as a potential mediator

The belief in a just world (BJW) refers to an individual's firm conviction that the world is fair and orderly, where people receive what they deserve and are placed where they belong (Lerner and Miller, 1978). As a stable cognitive schema, BJW provides individuals with a meaning-making framework for understanding social events, reinforcing their conviction that good deeds will ultimately be rewarded and wrongdoings will inevitably face punishment. This mechanism sustains a sense of controllability and predictability regarding the world (Lerner, 1980). According to social cognitive theory, an individual's self-cognition profoundly influences their beliefs and interpretations of the external environment (Bandura, 1986). Self-connection, defined as an individual's clear awareness of intrinsic authentic feelings and values (Zhao et al., 2024), constitutes the cognitive foundation for interpreting the external world. Individuals with high levels of self-connection often possess a clearer self-concept and a more stable internal value system. This psychological characteristic renders them more inclined to endorse the fairness of the world, as BJW is closely associated with an individual's meaning-making system (Wang, 2022; Dalbert, 2013). Furthermore, the psychological resilience resources afforded by self-connection may enhance individuals' capacity to buffer against unjust events, enabling them to maintain fundamental trust in the fairness of the world even when confronted with negative information in online environments (Liu et al., 2025).

According to justice motivation theory, BJW is not merely a static cognitive belief but also serves as a significant motivational force driving individuals to take action aimed at restoring a sense of justice (Lerner and Miller, 1978). Cognitive dissonance theory posits that when individuals encounter or witness unjust events, their BJW is threatened, leading to psychological distress that subsequently motivates them to restore or maintain the perception of a fair world through prosocial behaviors (Lerner, 1980). Furthermore, individuals with strong BJW are more inclined to believe that prosocial behaviors can yield positive long-term returns. This expectation reduces perceived uncertainty in online interactions, thereby enhancing their willingness to engage in online helping behaviors (Lerner, 1980; Li et al., 2022). Particularly in networked environments, where anonymity and the absence of non-verbal cues may weaken individuals' empathetic responses, BJW—as a stable cognitive resource—can provide behavioral motivation that transcends immediate emotional reactions (Christoff, 2014). Empirical studies also demonstrate that BJW not only directly predicts online prosocial behavior but also promotes positive actions in digital environments through mediating mechanisms such as enhanced psychological resilience and social self-efficacy (Liu et al., 2025; Yang et al., 2025). Thus, we propose the second hypothesis of this study: BJW mediates the relationship between self-connection and online prosocial behavior.

### 1.3 Meaning in life as a potential mediator

Meaning in life refers to the subjective psychological state in which individuals experience comprehension, purpose, and mattering in their existence (King and Hicks, 2021). Self-determination theory posits that the fulfillment of needs for competence, relatedness, and autonomy fosters intrinsic positive motivation, thereby prompting individuals to actively seek life meaning (Deci and Ryan, 2000). Self-connection, as a psychological state that facilitates deep engagement between individuals and their authentic selves, enables clear self-awareness and internal consistency. This sense of internal integration helps individuals perceive and construct meaning and purpose in life (Kernis and Goldman, 2006). When individuals experience high levels of self-connection, they are more likely to actively explore and experience meaning in life, as authentic self-awareness enables them to more readily identify goals and directions aligned with their personal values (Klussman et al., 2022a,b). Based on this, the present study hypothesizes that self-connection significantly and positively predicts meaning in life.

Meaning in life significantly influences individuals' social behaviors (Espinosa et al., 2022). Baumeister et al. (2013) noted that when individuals exhibit a stronger tendency to pursue meaning, they are more likely to position themselves as givers, willing to provide assistance to others and contribute to society. (Chang and Xie, 2018) also demonstrated that individuals with a stronger orientation toward meaning in life exhibit more pronounced altruistic tendencies. Previous studies have confirmed that meaning in life positively predicts prosocial behavior, that is, individuals with higher levels of meaning in life are more inclined to exhibit prosocial behaviors such as helping, sharing, and caring in daily life (Wang et al., 2018; Li et al., 2020). In networked environments, strong meaning in life can motivate individuals to transcend egocentric needs, prompting them to shift their focus toward the wellbeing of others. Consequently, this leads to increased prosocial behaviors in cyberspace, such as online mutual assistance, digital support, and knowledge contribution (Martela and Ryan, 2016; Guo et al., 2025). Moreover, meaning in life not only provides existential security and emotional coherence, reducing defensive self-focus and thereby freeing cognitive and emotional resources for social interaction (Heine et al., 2006; King and Hicks, 2021), but also expands the temporal integration horizon, enhancing individuals' willingness to invest in prosocial behaviors that offer delayed rewards (Carstensen, 2006). Therefore, this study hypothesizes that meaning in life positively predicts online prosocial behavior. This aligns with the fundamental tenet of self-determination theory, which posits that the fulfillment of intrinsic psychological needs drives positive social functioning by enhancing experiences of meaning in life (Klein, 2019). In conclusion, we propose the third hypothesis of this study: meaning in life mediates the relationship between self-connection and online prosocial behavior.

### 1.4 The chain mediating roles of belief in a just world and meaning in life

According to the integrative model of meaning in life, individuals' cognitive and emotional interpretations of life experiences are interwoven, jointly shaping their perception and pursuit of meaning in life (Park, 2010). BJW, as a stable cognitive schema, provides individuals with an expectancy framework of “good being rewarded.” This trust in world order further facilitates individuals' positive construction of life purpose and values, significantly enhancing their meaning in life (Huang et al., 2025; Janoff-Bulman, 1989). Previous studies have demonstrated that BJW facilitates individuals' integrative understanding of the external world (Otto and Schmidt, 2007), promotes positive emotional experiences (Bulman and Wortman, 1977), and thereby enhances intrinsic motivation for pursuing long-term goals (Meng et al., 2019; Yi et al., 2019; Chen et al., 2023). These factors collectively constitute critical antecedents of meaning in life. Building on previous research, the present study further hypothesizes that individuals with high levels of self-connection in online contexts exhibit stronger self-efficacy and social responsibility, are more likely to internalize and sustain positive BJW, and that such belief subsequently facilitates the formation of meaning in life, ultimately driving prosocial behaviors in online environments. Based on this, we propose the fourth hypothesis of this study: BJW and meaning in life play chain mediating roles in the relationship between self-connection and online prosocial behavior.

**Figure 1:**
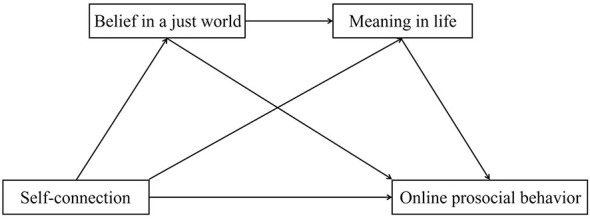
Hypothesis model diagram.

In conclusion, this study will investigate the relationship between self-connection and online prosocial behavior among college students, as well as the chain-mediated roles of BJW and meaning in life (see the Hypothesis Model in Figure 1).

## 2 Method

### 2.1 Participants

The study focused on full-time undergraduate and graduate students from two universities in Sichuan Province, employing a convenience sampling method. A total of 1,450 questionnaires were distributed. After excluding responses with repetitive patterns and excessive missing data, 1,303 valid questionnaires were retained, yielding a 89.86% valid response rate. The sample comprised 625 male students (48.00%) and 678 female students (52.00%); 233 freshmen (17.90%), 318 sophomores (24.40%), 410 juniors (31.50%), 274 seniors (21.00%), and 68 graduate students (5.20%). Participants ranged in age from 18 to 26 years (*M* = 20.94, SD = 1.60).

### 2.2 Measures

#### 2.2.1 Self-connection scale

This study employed the Self-Connection Scale developed by Klussman et al. (2022a,b), which comprises 12 items across three dimensions: self-awareness, self-acceptance, and self-consistency. The scale uses a 7-point rating system, with higher scores indicating stronger self-connection. The Chinese version of this scale has demonstrated good psychometric properties among college students (Zhao et al., 2024). In the present study, the internal consistency coefficient was 0.87.

#### 2.2.2 Belief in a just world scale

This study employed the Belief in a Just World Scale revised by Su et al. (2012), which comprises two dimensions—personal belief in a just world and general belief in a just world—totaling 13 items. The scale uses a 6-point rating system, with higher scores indicating stronger BJW. The internal consistency coefficient for this scale in the present study was 0.95.

#### 2.2.3 Meaning in life scale

This study employed the Meaning in Life Scale revised by Wang (2013), which comprises 9 items across two dimensions: meaning existence and meaning pursuit, assessing individuals' perceived presence of meaning and their motivation to seek meaning, respectively. The scale uses a 7-point rating system, with higher scores indicating greater meaning in life. In the present study, the internal consistency coefficient was 0.85.

#### 2.2.4 Online prosocial behavior scale

This study employed the Online Prosocial Behavior Scale developed by Zhang et al. (2021), which comprises 16 items across four dimensions: online helping, online altruism, online reciprocity, and online cooperation. The scale uses a 4-point rating system, with higher scores indicating stronger tendencies toward online prosocial behavior. In the present study, the internal consistency coefficient was 0.94.

## 3 Results

### 3.1 Common method variance test

Given that this study relied on self-report questionnaires, common method variance (CMV) may pose a potential threat to validity. To assess this, Harman's single-factor test was conducted. The results indicated that the first factor accounted for 30.48% of the variance (below the 40% threshold), suggesting that CMV was not a significant concern in this study and that the data were reliable.

### 3.2 Descriptive statistics and correlation analysis

Correlation analysis revealed significant associations among all variables (see Table 1). Self-connection was positively correlated with BJW, meaning in life, and online prosocial behavior. BJW was positively correlated with both meaning in life and online prosocial behavior. Meaning in life was also positively correlated with online prosocial behavior.

**Table 1:** Descriptive statistics and correlation analysis results (*N* = 1,303).

Variables	*M*	SD	1	2	3	4
1 Self-connection	4.73	0.83	1			
2 BJW	4.09	0.92	0.58^**^	1		
3 Meaning in life	4.80	0.92	0.49^**^	0.46^**^	1	
4 Online prosocial behavior	2.53	0.61	0.47^**^	0.53^**^	0.39^**^	1

^**^p < 0.01, ^***^p < 0.001.

### 3.3 Chain mediation model

**Figure 2:**
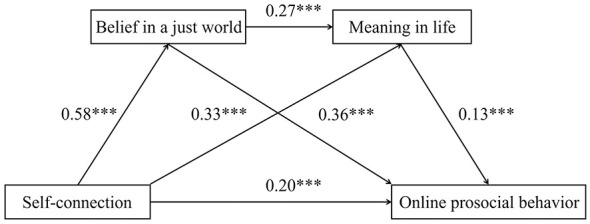
Chain mediation model of BJW and meaning in life. ***p < 0.001.

A chain mediation model was tested using Model 6 of the PROCESS macro (Hayes, 2018), with self-connection as the independent variable, online prosocial behavior as the dependent variable, and BJW and meaning in life as sequential mediators. The relationships among variables are presented in Table 2 and Figure 2. Regression analysis results indicated that self-connection significantly and positively predicted online prosocial behavior (β = 0.47, *p* < 0.001). Additionally, self-connection significantly and positively predicted both BJW (β = 0.58, *p* < 0.001) and meaning in life (β = 0.33, *p* < 0.001). Both BJW (β = 0.36, *p* < 0.001) and meaning in life (β = 0.13, *p* < 0.001) significantly and positively predicted online prosocial behavior. Furthermore, BJW significantly and positively predicted meaning in life (β = 0.27, *p* < 0.001).

**Table 2:** Regression analysis of variables in the chain mediation model (*N* = 1,303).

Variable	Online prosocial behavior		BJW		Meaning in life		Online prosocial behavior	
	β	***t***	β	***t***	β	***t***	β	***t***
Self-connection	0.47	19.01^***^	0.58	25.43^***^	0.33	11.56^***^	0.20	6.73^***^
BJW					0.27	9.52^***^	0.36	12.39^***^
Meaning in life							0.13	4.99^***^
*R^2^*	0.22		0.33		0.29		0.33	
*F*	361.47^***^		646.80^***^		262.73^***^		216.02^***^	

All variables in the model are brought back into the equation after standardized processing. ^***^p < 0.001.

Mediation effects were tested using the bootstrap method with 5,000 resamples. Results are presented in Table 3. The total effect was 0.45. The direct effect of self-connection on online prosocial behavior was significant [β = 0.18, 95% CI (0.12,0.24)]. The total indirect effect through BJW and meaning in life was significant [β = 0.27, 95% CI (0.23,0.32)], accounting for 60.00% of the total effect. Specifically, the indirect effect through BJW was significant [β = 0.21, 95% CI (0.17,0.25)], accounting for 46.67% of the total effect; the indirect effect through meaning in life was significant [β = 0.05, 95% CI (0.03,0.07)], accounting for 11.11%. The chain mediation effect via BJW and meaning in life was also significant [β = 0.02, 95% CI (0.01,0.03)], accounting for 4.44% of the total effect.

**Table 3:** Chain mediation effects of BJW and meaning in life (*N* = 1,303).

Path	Effect size	Boot SE	95% CI		Relative mediation effect proportion
			Lower	Upper	
Total effect	0.45	0.25	0.40	0.50	
Direct effect	0.18	0.29	0.12	0.24	
Total indirect effect	0.27	0.22	0.23	0.32	60.00%
Indirect effect 1	0.21	0.02	0.17	0.25	46.67%
Indirect effect 2	0.05	0.01	0.03	0.07	11.11%
Indirect effect 3	0.02	0.01	0.01	0.03	4.44%

Indirect effect 1: self-connection → BJW → online prosocial behavior; indirect effect 2: self-connection → meaning in life → online prosocial behavior; indirect effect 3: self-connection → BJW → meaning in life → online prosocial behavior.

## 4 Discussion

This study examined full-time undergraduate and graduate students from two universities in Sichuan Province to investigate the relationship between self-connection and online prosocial behavior, as well as the chain-mediation effects of BJW and meaning in life. The results indicated that self-connection significantly predicted online prosocial behavior. Specifically, college students with higher levels of self-connection were more likely to engage in prosocial behaviors in online environments. Further mediation analyses revealed that self-connection was associated with online prosocial behavior not only indirectly through BJW and meaning in life separately, but also sequentially through the chain-mediation pathway of BJW and meaning in life.

### 4.1 Self-connection and online prosocial behavior

The study found that self-connection significantly predicted college students' online prosocial behavior, thereby supporting Hypothesis 1. According to the definition proposed by Klussman et al. (2022a,b), self-connection encompasses three core dimensions: self-awareness, self-acceptance, and self-consistency. College students with high levels of self-connection typically exhibit clearer self-concepts. Sun et al. (2026) found that individuals with higher self-concept clarity, by virtue of stable and clear self-perceptions, experience reduced anxiety regarding resource scarcity and engage less in upward social comparison, thereby demonstrating greater willingness to exhibit prosocial behaviors. Furthermore, individuals with high levels of self-connection exhibit greater congruence between intrinsic values and extrinsic behavioral manifestations, which provides a stable psychological foundation for the emergence of prosocial behavior (Klussman et al., 2022a,b). According to Self-Determination Theory, when individuals establish connection with their authentic selves, their need for autonomy is satisfied, enabling them to make behavioral choices based on their genuine preferences rather than external pressures. This positive psychological resource can further stimulate autonomous prosocial motivation (Deci and Ryan, 2000). Given the anonymity and weak social constraints characteristic of cyberspace, individuals can actively exhibit prosocial behaviors only when they possess strong intrinsic motivation (Hao et al., 2025). Moreover, individuals who maintain connection with their core selves exhibit reduced defensive behaviors and increased openness when facing external threats (Klussman et al., 2022a,b; Sherman and Cohen, 2006). In online environments, self-connection may enhance intrinsic security and self-affirmation, thereby reducing defensive responses and fostering greater willingness to offer assistance to others. In conclusion, self-connection significantly predicts college students' online prosocial behavior: those with higher levels of self-connection are more likely to engage in prosocial behaviors in online environments.

### 4.2 The mediating effect of belief in a just world

The results indicated that BJW mediated the relationship between self-connection and online prosocial behavior, thereby supporting Hypothesis 2. Specifically, self-connection was associated with the expression of online prosocial behavior among college students by maintaining BJW, consistent with previous research (Correia et al., 2009). College students with higher levels of self-connection exhibit clearer self-concepts, along with elevated self-efficacy and self-esteem (Dalbert, 2013). These factors contribute to the formation and maintenance of fundamental beliefs in a just and orderly world (Lerner, 1980). Social cognitive theory also posits that individuals' self-cognition influences their beliefs and interpretations of the external world (Bandura, 1986). Furthermore, BJW enhances individuals' sense of predictability and control over the world, making them more inclined to adhere to social norms, trust others, and exhibit prosocial behaviors (Bègue, 2002). Cognitive dissonance theory further supports this perspective. When confronted with unjust incidents occurring online, college students holding BJW experience cognitive dissonance, leading to psychological distress that subsequently motivates them to engage in prosocial behaviors to restore or maintain their belief that the world is just and orderly (Lerner, 1980). Additionally, college students with high BJW are more inclined to believe that prosocial behaviors yield rewards. This expectation reduces perceived uncertainty in online interactions and enhances their willingness to engage in prosocial behaviors (Lerner, 1980; Li et al., 2022; Yang et al., 2025). However, the behavioral implications of BJW are profoundly context-dependent and do not invariably translate into positive outcomes. Prior research has demonstrated that when individuals encounter entrenched or seemingly unchangeable injustice, BJW may elicit adverse social effects through mechanisms such as victim blaming and the rationalization of existing inequalities (Lerner, 1980; Hafer and Begue, 2005). The positive mediating effect of BJW observed in the present study may be attributable to the fact that the online prosocial behavior context offers individuals a constructive pathway to restore their sense of justice through active helping, thereby mitigating the likelihood of defensive rationalization. In conclusion, this study reveals the intrinsic mechanism by which self-connection is associated with online prosocial behavior: self-connection is associated with BJW, thereby contributing to the expression of online prosocial behavior among college students.

### 4.3 The mediating effect of meaning in life

The results revealed that meaning in life mediated the association between self-connection and online prosocial behavior, providing support for Hypothesis 3. This finding elucidates an underlying psychological mechanism through which self-connection is associated with college students' online prosocial behavior. According to Self-Determination Theory, the satisfaction of fundamental psychological needs—namely competence, relatedness, and autonomy—cultivates intrinsic motivation that fosters the pursuit of meaning in life (Deci and Ryan, 2000). Self-connection appears to satisfy the basic psychological needs for autonomy and relatedness, thereby establishing a foundational basis for the development of meaning in life. Furthermore, individuals with heightened self-connection demonstrate clarity regarding their personal values and life goals, which facilitates a more accessible experience of meaning in life (Klussman et al., 2022a,b). As an intrinsic motivational force, meaning in life propels individuals to transcend self-interest and extend their concern beyond egocentric perspectives toward the welfare of others (Baumeister et al., 2013). Prior research has established that meaning in life positively predicts prosocial behaviors in offline contexts (Wang et al., 2018; Li et al., 2020), and the present findings extend this relationship to digital environments. College students who experience greater meaning in life are more inclined to perceive prosocial behaviors—such as information sharing and emotional support—as expressions of self-worth, consequently exhibiting increased online prosocial behavior. Moreover, affective dynamics inherent to digital environments may shape prosocial behavior; individuals with elevated meaning in life are more likely to recognize others' needs through empathy mechanisms, thereby facilitating supportive interactions (Luo et al., 2025). This pattern aligns with the Meaning Maintenance Model, which posits that individuals construct meaning in life through coherent self-world understanding and subsequently utilize this meaning to guide social behavior (Heine et al., 2006). Collectively, these findings suggest that self-connection may contribute to online prosocial behavior through the enhancement of meaning in life.

### 4.4 Chain mediating effects of belief in a just world and meaning in life

The results indicated that BJW and meaning in life sequentially mediated the relationship between self-connection and online prosocial behavior, providing support for Hypothesis 4. This finding elucidates the pathway mechanism through which self-connection is associated with online prosocial behavior: self-connection may contribute to individuals' BJW, which in turn elevates their meaning in life, ultimately contributing to the expression of online prosocial behavior.

Regarding the association between BJW and meaning in life, the results revealed that BJW significantly and positively predicted meaning in life, consistent with prior research (Tian et al., 2024; Chen, 2021). As a stable cognitive schema, BJW imbues individuals with a sense of certainty and perceived control over the external environment (Bègue, 2002), enabling them to maintain psychological stability and goal-directed focus even under adverse conditions (Van Tilburg and Igou, 2019). This cognitive framework provides a foundational basis for deriving meaning from life experiences (King and Hicks, 2021). Extant research has demonstrated that BJW substantially enhances the formation of meaning in life by strengthening individuals' sense of control and perceived order (Wu, 2024). Furthermore, the integrative model of meaning in life posits that individuals' cognitive interpretations of the external world and their emotional experiences are inextricably intertwined, jointly shaping their perception and pursuit of meaning in life (King and Hicks, 2021). In contexts characterized by elevated online anonymity, individuals confront heightened uncertainty and perceived threat. Under such conditions, BJW functions as a psychological buffer; by maintaining the conviction that effort yields proportionate rewards, individuals can withstand environmental uncertainties and thereby safeguard their meaning in life from erosion. This pattern aligns with the core tenets of the Meaning Maintenance Model, which proposes that individuals construct meaning through coherent understandings of themselves, the world, and their interrelations, subsequently utilizing this meaning to guide social behavior (Heine et al., 2006). Within this process, BJW operates both as an outward extension of self-connection and as a cognitive foundation for the development of meaning in life, ultimately contributing to the expression of online prosocial behavior among college students. However, the serial mediation effect through BJW and meaning in life explained only a modest portion of the total effect, suggesting that this pathway is unlikely to represent the primary mechanism. Future research should therefore examine this mechanism alongside other potential mediators to more fully elucidate the relationship between self-connection and online prosocial behavior.

In conclusion, this study demonstrates the serial mediating roles of BJW and meaning in life in the relationship between self-connection and online prosocial behavior. By systematically examining four dimensions—self-cognition, world cognition, meaning construction, and behavioral expression—we elucidated the underlying mechanisms through which self-connection is associated with online prosocial behavior. This research not only advances theoretical understanding of the developmental processes underlying online prosocial behavior but also identifies potential targets for future intervention efforts.

## 5 Limitations and future research

The present study examined the relationship between self-connection and online prosocial behavior and elucidated its underlying psychological mechanisms, thereby offering several theoretical contributions. Nevertheless, the following limitations should be acknowledged, and these issues warrant attention in future research endeavors.

First, the present study was limited by sample representativeness. The sample comprised exclusively full-time undergraduate and graduate students recruited from two universities in Sichuan Province, exhibiting relatively homogeneous geographical distribution and demographic characteristics. These constraints may limit the generalizability of the findings. Individuals from different regions or age groups may demonstrate variability in self-connection, BJW, meaning in life, and online prosocial behavior. Future research should expand the sampling scope to encompass universities from diverse geographical regions and institutional types, as well as broader youth populations, thereby enhancing the external validity of the findings.

Second, the study was limited by its cross-sectional design. The research employed a single-timepoint questionnaire survey, precluding rigorous causal inferences regarding the relationships among variables. Although a serial mediation model was theoretically specified linking self-connection, BJW, meaning in life, and online prosocial behavior, cross-sectional data cannot exclude the possibility of reverse causality or confounding by unmeasured third variables. For instance, BJW may foster the integration of one's self-concept by providing cognitive security. That is, when individuals believe the world is fair, their past experiences and self-narratives are more likely to form a coherent whole, thereby enhancing self-connection (Lerner, 1980). Additionally, online prosocial behavior may, in turn, enhance meaning in life by satisfying needs for competence and relatedness (Martela and Ryan, 2016). In online contexts, helpers often receive immediate feedback and social recognition through digital platforms; such experiences of need satisfaction may be particularly salient, thereby strengthening their meaning in life. Future research should employ longitudinal designs or experimental methodologies to further validate the hypothesized causal directions through multi-timepoint data collection or direct variable manipulation.

Third, the study was limited by the exclusive reliance on self-report measures. All variables were assessed using self-report questionnaires. Although statistical tests indicated that common method bias remained within acceptable limits, data derived from a single source may nonetheless inflate observed correlations among variables. Furthermore, as online prosocial behavior carries inherent social desirability, participants may have provided socially desirable rather than accurate self-reports, introducing systematic positive bias that threatens the construct validity of the findings. Future research should integrate multiple assessment modalities, such as peer ratings, behavioral experiments, or online behavioral trace data, thereby enhancing the objectivity and accuracy of data collection.

Fourth, the study was limited in its examination of intermediary mechanisms. The research exclusively investigated the serial mediating effects of BJW and meaning in life, without considering potential mediating or moderating variables. For instance, individual personality traits, internet use motivations, or perceived social support may influence the strength of the relationship between self-connection and online prosocial behavior among college students. In addition, empathy and social responsibility may represent two promising alternative pathways. Individuals with higher levels of self-connection may be more inclined to engage in online prosocial behavior not only because they perceive the world as meaningful and just, but also because they experience greater empathic concern for others or feel a stronger sense of responsibility to contribute to collective wellbeing. Future research should systematically examine these and other potential mediators and moderators to construct moderated mediation models, thereby further elucidating the boundary conditions of these mechanisms.

In conclusion, future research should address the aforementioned limitations by improving sample representativeness, adopting longitudinal or experimental designs, diversifying assessment modalities, examining moderated mediation mechanisms, and refining behavioral typologies. Such efforts would yield a more comprehensive and nuanced understanding of the relationship between self-connection and online prosocial behavior and its underlying psychological mechanisms.

## Data Availability

The raw data supporting the conclusions of this article will be made available by the authors, without undue reservation.
